# Revealing fine scale subpopulation structure in the Vietnamese H'mong cattle breed for conservation purposes

**DOI:** 10.1186/1471-2156-11-45

**Published:** 2010-06-07

**Authors:** C Berthouly, JC Maillard, L Pham Doan, T Nhu Van, B Bed'Hom, G Leroy, H Hoang Thanh, D Laloë, N Bruneau, C Vu Chi, V Nguyen Dang, E Verrier, X Rognon

**Affiliations:** 1 CIRAD, UPR AGIRs, Campus International de Baillarguet, 34398 Montpellier, France; 2 AgroParisTech, UMR1313, Génétique Animale et Biologie Intégrative, F-75005 Paris, France; 3 INRA, UMR1313 Génétique Animale et Biologie Intégrative, F-78350 Jouy-en-Josas, France; 4 NIAH, Tu Liem, Ha Noi, Vietnam

## Abstract

**Background:**

During the last decades, there has been an acceleration of the loss of domestic animal biodiversity. For conservation purposes, the genetic diversity of the H'Mong cattle, an indigenous local breed was studied. Single-nucleotide polymorphisms (SNP) of the *SRY *gene and mtDNA D-Loop sequence were analysed to clarify the origin of the breed. The genetic diversity was assessed through genetic data with twenty-five FAO microsatellites, and morphometric data with five body measurements from 408 animals sampled from eight districts of the Ha Giang province.

**Results:**

The *SRY *genes were all of the zebu type. Among the 27 mtDNA haplotypes, 12 haplotypes were of the taurine type and the remaining 15 of the zebu type. This indicates female taurine introgression in the zebu H'Mong. The observed and expected heterozygosity ranged from 0.616 to 0.673 and from 0.681 to 0.729 respectively according to district, with low genetic differentiation (F_ST _= 0.0076). Multivariate analysis on morphometric and genetic data shows a separation of districts into two groups following a south-west/north-east cline and admixture analysis confirmed the two clusters, but no differentiation of taurine introgression between clusters was observed. A possible admixture with the Yellow cattle breed from a neighbouring province was suggested through genetic data and householder interviews.

**Conclusions:**

In this study we demonstrate the interest of fine-scale sampling for the study of genetic structure of local breeds. Such a study allows avoiding erroneous conservation policies and on the contrary, proposes measures for conserving and limiting crossbreeding between the H'Mong and the Yellow cattle breeds.

## Background

A total of 990 cattle breeds have been reported throughout the world, and 897 are classified as local or indigenous breeds and 93 as transboundary breeds. Among the 258 breeds reported in Asia, 11% are classified at risk, 51% are not in danger, whereas the status of the remaining breeds (38%) is unknown (Scherf [1]). Asian cattle can be subdivided into humped and humpless cattle. The humped zebu breeds are more prevalent in southern regions of Asia, particularly in India and Pakistan. Humpless taurine cattle are found across most of the Asian continent in the northern regions. Chinese cattle are subdivided into three groups: the Turano-Mongolian type above the Yellow river, which is of the taurine type, the Changzhu type below the Yangtze River which is of the zebu type, and a third type in the intermediary central area of Huanghuai which is a hybrid of taurine and zebu breeds.

In Vietnam, 22 cattle breeds are currently present including 4 breeds identified as local breeds [2]: the Bo U Dau Riu in the southeastern area of Hanoï; 2) the Bo Vang (Yellow cattle) in the western and southern provinces around Hanoï; 3) the Chau Doc in the Mekong Delta; and 4) the H'mong breed in the northern provinces bordering China and more specifically in the Ha Giang province.

Little information on breed description has been generated. Almost all Vietnamese breeds have a uniform coat colour varying to yellow from red froment, except for the H'mong breed for which black colour is also common. Also, the shape of the hump has been used in Vietnam as a criterion for breed differentiation. According to the "Atlas of Vietnamese Breeds" [2], the Yellow cattle breed seems to have a "hump like Indian type in opposition to the Chinese type" and in this breed the hump is also smaller. The Bo U Dau Riu (U meaning Hump) is named such because of its specific black and yellow hump. The H'mong breed, besides being the only breed with a black coat, has a Chinese cattle type hump which is also less developed and sloping down in the front.

This breed also has the singularity to be linked with a specific ethnic group: the H'mong people.

The H'Mong people originate from the Huanghuai area in China, they first entered Vietnam by the Ha Giang province and continued their migration south through Thailand, mixing their cattle breeds with local ones.

No genetic information is known about this breed, but considering its origin probably in southern China and the genetic studies on Chinese breeds [3,4], H'Mong cattle is assumed to be of the taurine-zebu hybrid type. This breed is raised in a province with a sharp variation in altitude subdivided into many valleys. As a consequence, travelling from village to village is very difficult and can mainly only be done by walking. In addition, the H'Mong ethnic group used to live in regions located 1000 meters above sea level or higher, which made them semi-isolated and disrupted the distribution of cattle limiting them to the two mountainous areas of the province. As agropastoralists, H'Mong householders raise cattle for draught power in a free-foraging way without any selection or breeding management. Geographical characteristics and farming practices may have structured the cattle population. Assessing its genetic status is all the more important to design rational breeding strategies for its sustainable management.

The aims of this study were the following: (1) to confirm the taxonomic hybrid status of this breed using mtDNA sequences, the *SRY *gene and microsatellites; (2) and to analyse the genetic structure of the H'Mong cattle breed through phenotypes and genotypes in combination with a fine-scale survey. Management practices were also studied to ascertain the relevance of the biological results.

## Methods

### Sampling procedure

**Figure 1:**
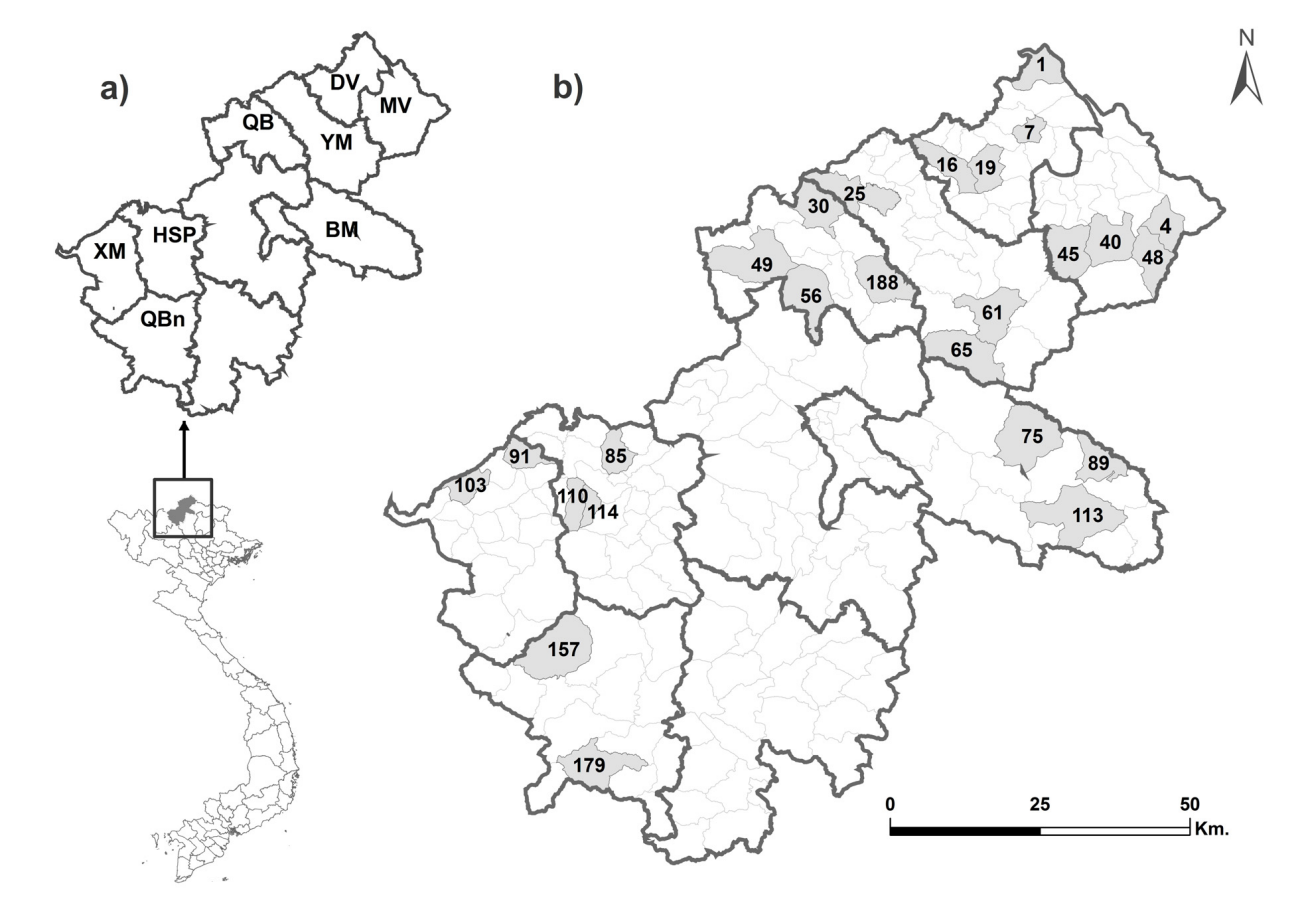
**Map of the Ha Giang province: a) district borders (bold lines), b) commune sampled in grey light**. See Table 1 for district codes.

The Ha Giang province is a Vietnamese province bordering China *(22°08-23°19'N; 104°33'-105°33'E)*. We sampled 8 districts among the 11 districts that are represented by 25 communes, leading to a total of 133 villages (Figure 1). For each of the 407 samples (Additional File 1 &2), both measurements and genotypes were recorded in a database linking morphometric information to molecular information.

### Householder interviews

Householders were questioned about their knowledge of the pedigree of each animal and their origin. Eight categories of origin were considered: Farm; Village; Commune; District; District Market; Other district; Outside the Province; Project (many poverty alleviation projects have given cattle to householders with no information about their origin).

### Molecular markers

Genomic DNA was extracted from tissue samples using the QIAamp Kit from QIAGEN.

### Monoparental markers

Genotyping of SNP at position 641 of the SRY gene following Kikkawa *et al. *[5] was conducted by pyrosequencing (see Addtional File 1). The 145 bulls from Ha Giang were analysed jointly with the Gascon taurine (3) and Arab zebu breed (3) from Chad, which were used as controls.

The complete mtDNA D-loop region was amplified according to Loftus *et al. *[6] from 123 H'Mong cattle (see supplementary data). Sequences from GenBank from 23 breeds were used and organised into four groups: the European taurine, Chinese taurine, Chinese zebu and Chinese admixed group (Additional File 3). Reference mitochondrion sequences for *Bos taurus *(GenBank accession number AF492351; green ellipse, [7]) and *Bos indicus *(GenBank accession number AF492350; red ellipse, [8]) were included as controls in the analysis. The cattle mtDNA newly sequenced in this study have been deposited in GenBank under accession numbers FJ800840-FJ800962.

### Microsatellite markers

Genotypes were done in the laboratory of the National Institute of Animal Husbandry in Hanoi using the 30 FAO/ISAG microsatellites. However, technical difficulties were encountered for 5 markers leading to only 25 markers being used for analysis. Thirty control samples from the taurine breed (Gascon and Salers breeds) and African zebu with the international genotype calibration from CadBase were used at the same time and in the same conditions as the Vietnamese samples. This procedure allows standardising Vietnamese genotypes with the CadBase datasets available at http://www.projects.roslin.ac.uk/cdiv/. From this database, genotypes for 8 markers *(BM1818, ETH3, INRA063, HEL1, TGLA227, TGLA122, BM2113, INRA023) *were found to be in common with 7 relevant breeds including Asian taurine breeds: the Hanwoo Korean, the Chinese Yellow Yanbian breed and the Japanese Black breeds published by Kim *et al. *[9]. The set included 2 Indian breeds first analysed by Loftus *et al. *[10] and later completed by Ibeagha-Awemu *et al. *[11] who also published the German Simmental included in the dataset. The information about the origin of the Holstein used was not available (Table 1, Additional File 1). Genotypes of the H'mong breed are available upon request.

**Table 1:** Summary of breeds and Ha Giang district populations and their polymorphism measures.

Breeds	Origin	Type	N_i_	He	Ho	A	A_e_	*F*_*IS*_	*F*_*ST*_	*D*_*HWE*_	*fm*
Hanwoo (HAW)^a^	Korean taurus	taurus	91	0.751	**0.754**	**8.1**	4.0	-0.004	0.137		

Chinese Yanbian breed (CYC)^a^	Chinese taurus	taurus	41	0.748	0.732	7.2	4.0	0.023	*0.133*		

Japanese Black (JAB)^a^	Japanese taurus	taurus	36	*0.520*	*0.576*	*3.4*	*2.1*	*-0.110*	0.266		

Simmental (SIM)^b^	Germany	taurus	50	**0.757**	0.687	8.0	**4.1**	0.094	0.154		

Holstein (HOL)	Europe	taurus	150	0.720	0.725	7.2	3.6	-0.007	0.165		

Nellore (NEL)^b^	Indian zebu	zebu	27	0.634	0.631	5.6	2.7	0.020	0.194		

Ongole (ONG)^b^	Indian zebu	zebu	30	0.595	0.600	5.5	2.5	-0.009	**0.221**		

H'Mong (HG)	Vietnam	zebu	413	0.673	0.632	7.2	3.1	**0.061**	0.181		

Within Ha Giang province	Districts		*for 25 markers*								

H'Mong (HG)	Quang-Binh (QBn)		10	0,695	0,633	*4,7*	*2,90*	0,085	0,016	*0*	/
	
	Hoang-Su-Phi (HSP)		34	0,716	0,667	6,8	3,40	*0,07*	0,014	4	0,247
	
	Xin-Man (XM)		51	**0,729**	**0,673**	7,4	**3,60**	0,075	0,016	5	0,248
	
	Quan-Ba (QB)		54	0,724	0,63	7,4	3,50	**0,128**	0,011	**6**	*0,214*
	
	Yen-Minh (YM)		52	0,721	0,657	7,2	3,50	0,087	*0,007*	2	0,223
	
	Dong-Van (DV)		88	0,713	0,638	**7,7**	3,40	0,103	0,009	4	0,242
	
	Meo-Vac (MV)		82	0,703	0,634	7,5	3,30	0,095	0,013	4	0,244
	
	Bac-Me (BM)		32	*0,681*	*0,616*	6,7	3,00	0,093	**0,02**	3	**0,285**

N_i_: Number of individuals; A: mean number of alleles; A_e_: effective number of alleles; D_HWE_: number of alleles in heterozygote deficiency; *fm*: molecular coancestry; ^a ^published by [6], ^b ^published [7] and [9]

**in bold**: maximum values, *in italic*: minimum values

### Morphological data

All animals genotyped were adults. Five measurements were taken for each animal: thorax depth, height at withers (*HW*), body length (*BL*), heart girth (*HG*) and ear length (*EL*). These measures were combined into 4 indexes: the index of slenderness *IGs *according to Lauvergne & Souvenir Zafindrajaona [12], the body length index *I*_*BL *_= *BL/HW*, the heart girth index *I*_*HG *_= *HG/HW *and the Ear index *I*_*EL *_= *EL/HW*.

### Statistical analysis

#### Molecular diversity, within and between populations

##### mtDNA marker

Haplotype and nucleotide diversity and minimum spanning network were computed using Arlequin 3.1 [13]. We constructed an unrooted neighbor-joining (NJ) tree of all sequences under the Tajima & Nei model using MEGA 4.1 software [14].

##### Microsatellite markers

The presence of null alleles was tested using FreeNA [15]: loci with estimated frequencies of null alleles *r *= 0.2 were considered to be potentially problematic for calculations. For the H'Mong population, districts were considered as sampling units for performing preliminary estimations of genetic polymorphism. Genetic polymorphism measures were calculated using GENETIX 4.4 [16]. The GENEPOP software [17] was used to compute *F-statistics *[18] and the Hardy-Weinberg equilibrium test [17]. Test significance was corrected with sequential Bonferroni correction on loci.

The matrix of Reynolds unweighted distances DR [19] was computed. Regarding the DR distances, a Neighbor-Joining tree was established with 1000 bootstraps on the loci. A DR matrix and Neighbor-Joining tree were computed using POPULATION v.1.2.28 (Olivier Langella; available at http://bioinformatics.org/project/?group_id=84)

We calculated the contributions (*c*_*i*_) of each population, which could maximise the total diversity at the next generation as proposed by Caballero & Toro [20].

We investigated the genetic structure and individual assignments using a Bayesian clustering procedure implemented in STRUCTURE [21], with the admixture model and correlated allele frequency [22] and 15 runs for *K *= 1-10, with 10^6 ^iterations following a burn-in period of 300,000. The values for the number of clusters (*K*) were assessed according to Evanno *et al. *[23] by the (*D.ΔK*) criterion. The programme estimates the posterior distribution (*q*) of each individual's admixture coefficient. The grid map of the admixture coefficient *q *was constructed using MAPINFO^®^.

### Morphometric Analysis

Morphological data were analysed using single trait linear hierarchical mixed models with the SAS^® ^software (see Berthouly *et al. *[24]). A linear discriminant analysis was used on the whole set of markers and measurements. Since this set contains a combination of quantitative and qualitative variables, we used an approach similar to that of Hill & Smith [25]. This approach is a combination of an internal correspondence analysis on markers (Cazes *et al. *[26]; Laloë *et al. *[27]) and of a principal component analysis on quantitative measures. This approach allows using a variable set and comparing the contribution of morphometry and genetics for the differentiation of populations. This approach is based on functions available in the ade4 package (Chessel *et al. *[28]; Dray & Dufour [29]) of the R software (R development core team [30]). For this analysis, we removed samples from the QBn district because of the low number of samples (9) compared to other districts.

## Results

### Marker analysis

#### Monoparental markers

From the SRY gene, all the bulls had the T base at the 641 position. This indicates that they all have a *Y *chromosome of *B. indicus *origin.

We obtained sequences of 825 bp of the mtDNA D-loop from 123 H'mong cattle, resulting in 27 haplotypes. A total of 53 polymorphic sites were observed involving 49 transitions and 5 transversions.

#### Biparental markers

Within the Ha Giang cattle dataset, no loci had *r *values higher than 0.2, therefore it was assumed that null alleles would not significantly bias the genetic estimates. A total of 195 alleles were detected. The observed heterozygosity per locus averaged 0.643 and ranged from 0.293 for locus HEL1 to 0.821 for locus HAUT27 (Additional File 4). According to the multiloci *F*_*ST*_, only 1.16% of the total genetic variability was explained by subdivision of populations among districts.

### Genetic relationships among breeds

**Figure 2:**
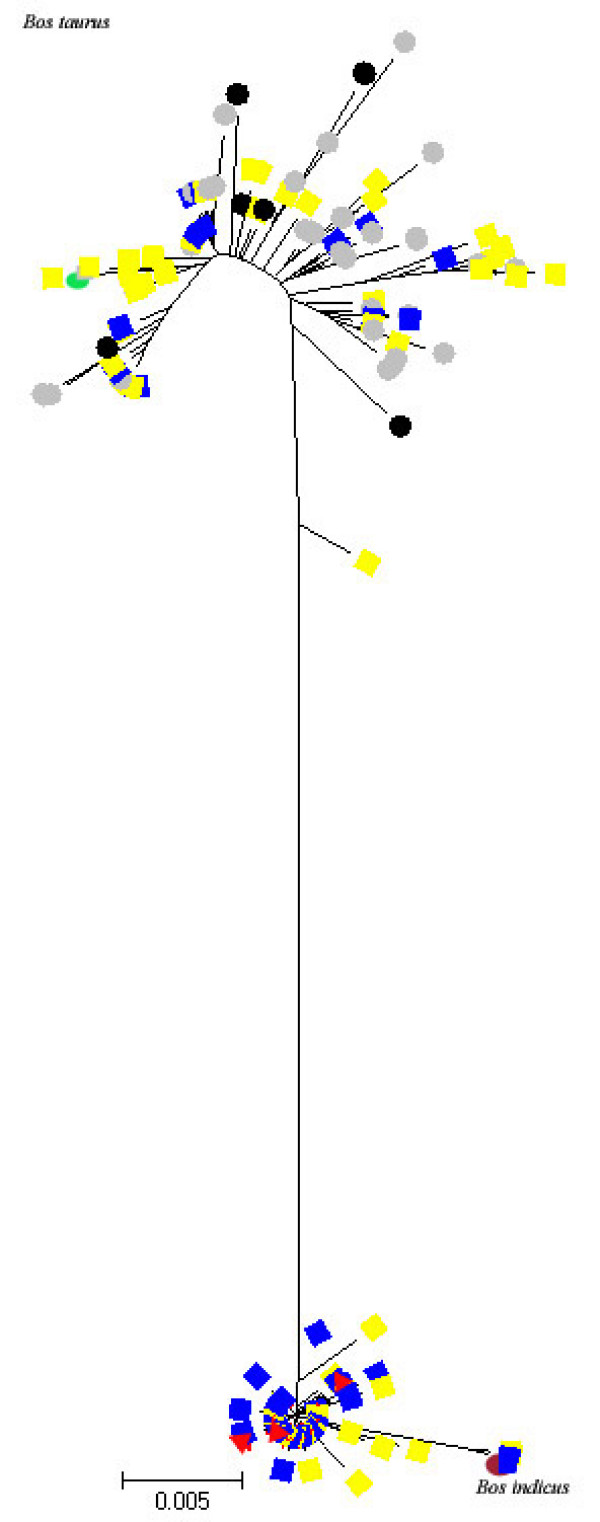
**Unrooted neighbour-joining tree of 304 cattle mtDNA D-loop sequences: European taurine breed (dark circle), Chinese taurine breed (grey circle), Admixed Chinese breed (Yellow square), HG population (dark square), Chinese zebu breed (red triangle)**.

For the mtDNA, the mean haplotype diversity was 0.860 ± 0.019 and 47.8% of the genetic diversity occurred between the four breed groups. The HG population showed the smallest F_ST _distance with the Chinese zebu group (0.173). The unrooted NJ tree of the 304 sequences (Figure 2) identified the two clades, taurine and zebu, with, on average, 40.78 nucleotide differences. The Chinese admixed breeds (Additional File 3) and the HG population fell into both clusters. Among the 123 H'Mong sequences, 96 sequences corresponding to 15 haplotypes belonged to the zebu clade while 27 animals belonged to the taurine clade (Figure 2). These 27 sequences constituted 12 haplotypes and were recognised as the taurine type.

**Figure 3:**
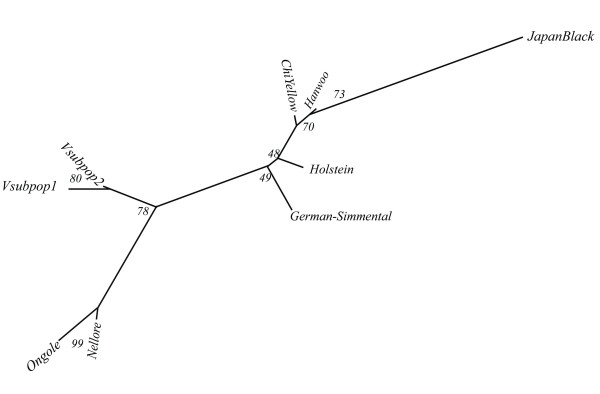
**Neighbour-joining tree of the 7 taurine and indicine breeds, and the two M'Hong subpopulations, based on DR distances and established with 1000 bootstraps on the loci (eight common microsatellites)**. Breed codes are given in Table 1.

The NJ tree showed a clear separation between the taurine and zebu breeds (Figure 3) using the 8 microsatellites in common between breeds from Table 1. The HG population is positioned between the Indian Zebu and European taurine breeds.

### The H'Mong cattle population

#### Within and between district diversity

There were, on average, 3.3 effective alleles per district (Table 1). The observed and expected heterozygosity averaged over all loci ranged from 0.616 to 0.673 and from 0.681 to 0.729 respectively. The average *F*_*IS *_in the Ha Giang province reached 0.092. Except for the QBn district, all district populations had more than two loci in heterozygote deficiency, and the maximum value of 6 loci deviated from HWE was reached for the QB district. The average molecular coancestry *f*_m _ranged from 0.214 in the QB district to 0.285 for the BM district corresponding to coancestry between full-sibs. According to the Caballero & Toro [20] procedure, a synthetic population that would maximise genetic diversity within the Ha Giang population would be composed of 70% of cattle from the QB district, followed by 27% from the YM district and the 3% remaining coming from the HSP district.

#### Morphometry and Multivariate analysis among district populations

Mean body traits between districts are summarised in Additional File 5. There were significant differences between males and females for all measures except for *EL *and *I*_*BL*_. Altitude had an effect on *HG *and *I*_*BL *_(data not shown). Pair-wise comparison between districts shows that only *HW *was significantly different after Bonferroni correction *(p-value < 0.005)*. Cattle from the MV district were significantly smaller than cattle from the three western districts: HSP, XM and QB *(p-value < 0.003)*. The smallest *HG *values were observed in the MV district for both sexes (134.2 cm; 142.4 cm) whereas the biggest females were observed in the XM district (144.0 cm) and males in the YM district (155.0 cm).

**Figure 4:**
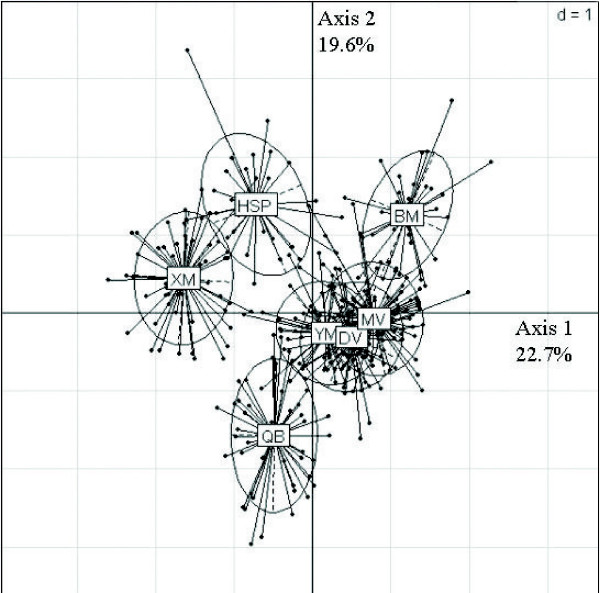
**Representation of the Ha Giang district H'Mong cattle populations using discriminant analysis (25 microsatellites + 5 morphometric traits) with the Hill & Smith procedure**. This procedure similar to PCA allows maximising differentiation between districts using genetic and morphometric data. The first axis coincides with an east/west cline and separates the districts into 2 groups. See Table 1 for district codes.

The first two axes of the discriminant analysis for the whole set of variates (25 markers + 5 measures) explained 22.7% and 19.6% of the observed inertia (Figure 4). The *HW *and *I*_*BL *_were the body measures with the highest contributions for discrimination (6.7% and 4.3% respectively). This procedure similar to PCA allows maximising differentiation between districts using genetic and morphometric data. The first axis coincides with an east/west cline and separates districts into 2 groups. The first group with bordering districts HSP and XM are positioned on the left; and the second one is on the right side with the eastern districts.

#### Subpopulation structure and gene flow

**Figure 5:**
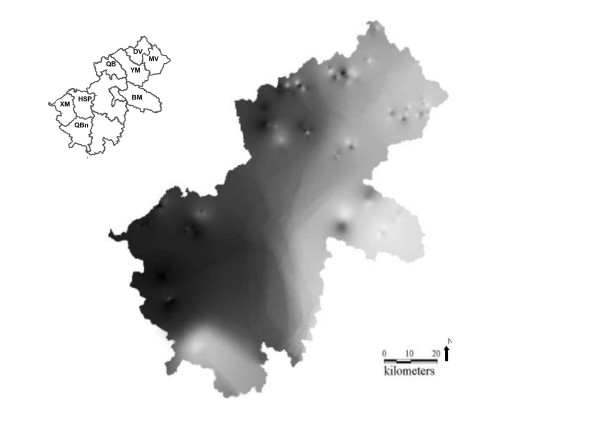
**Grid map of individual probabilities to belong to cluster 1 ranging from *q *< 0.03 in white to *q *> 0.96 in dark obtained by STRUCTURE using 25 microsatellites**.

The value of the criterion used (*D.ΔK*) was the highest for *K *= 2. This indicates that two sub-populations are the most likely figures for the cattle population. Communes were attributed to a preferential cluster with mean probabilities *(q) *ranging from 0.51 to 0.88 (Additional File 6). A grid map of individual probabilities of admixture to cluster 1 (*q*_1_) is represented in Figure 5. It is therefore possible to see that the admixture rate with cluster 1 is decreasing from the west to the east. The HSP and XM district had a mean admixture with cluster 1 of 0.77 and 0.83 respectively (Additional File 7). For the QBn district, commune 157 was strongly admixed with cluster 1 (0.86) and commune 179 with cluster 2 (0.73). Cluster 1 could be identified as the south-west province. Starting from DV, then MV and BM, we observed an increasing gradient of admixture with cluster 2, the maximum being observed in commune 113 with a mean admixture value of 0.88. Cluster 2 could be considered as the northeast province.

The comparison of morphometric traits assuming the two Bayesian genetic clusters shows that cattle from the southwest cluster (HSP, XM and QB) were about 5 cm significantly taller than cattle from the northeast cluster (Additional File 7).

AMOVA analysis using the mtDNA sequence, shows that only 0.003 of diversity occurred between clusters. The taurine and zebu haplotypes were found in both clusters (Additional file 8). No significant difference in taurine mtDNA admixture was observed between both clusters (Chi2 test; *p = 0.385*).

A total of 814 pedigree-origin interviews were done and the percentages of answers per category are summarised in Additional File 9. Householders from all but three communes kept and reproduced animals on their own farm, without reproduction management, with rates ranging from 2.2% to 46.7% in Family category. Exchanges occurred mostly within the commune but also with bordering communes. Nevertheless, in MV and DV districts, where two important cattle markets are held, the percentages of animals bought at district markets ranged from 3.1% to 64%. In commune 179 from the QBn district, nearly 67% of cattle were given by a development project (Additional File 9). This also happened but at a lower degree in communes 113 and 89 from the BM district. The BM district was also the one with the most important rate of cattle coming from outside the province (6.5% - 23.2%).

## Discussion

### Phylogenetic status

The H'Mong breed has yellow, red or black coat colour and a cervico-thoracic or thoracic hump [31]. Thus it seems to belong to the zebu group, but current zebu-like (i.e. humped) breeds are often the result of admixture between *B. indicus *and *B. taurus*, such as the Yunnan Yellow cattle or the Nayang Chinese breeds [3] even in the southeast and east Asian areas [32]. According to Cai *et al. *[3], cattle from south China have Y chromosomes of the zebu type while Lei *et al. *[32] found both taurine and zebu mtDNA, within the majority of the sampled breeds, suggesting maternal introgression of the taurine cattle. Similar results were obtained for the Ha Giang cattle population. The zebu-taurine introgression was also confirmed by the central position of the H'Mong population between both taurine and zebu clades in the NJ tree obtained using microsatellites.

Within the Ha Giang province, both subpopulations show similar rates of Taurine introgression. These results, and the pattern of taurine mtDNA distribution, allow us to assume that the hybridisation process preceded the genetic structuring into two subpopulations. Migratory records of the H'mong people and their livestock are in agreement with our results [33].

### Subpopulation structure

Within the H'Mong cattle population of the Ha Giang province, the multivariate procedure enlightens a southwest/northeast cline confirmed by the Bayesian approach. This genetic structure might result from two factors: (1) a geographic isolation with two mountainous areas. The two mountainous areas in the North (DV, MV, YM, QB districts) and in the South (XM, HSP districts) are separated by flatlands where no cattle are raised; (2) different origins of gene flow (neighbouring provinces, important cattle markets) are suggested from interview information. Indeed, 73.5% of the population from the Bac-Me district had a probability of more than 0.8 to belong to cluster 2. According to pedigree interviews, cattle from the three communes of the Bac-Me district were partly bought in from another province, and more specifically in the Cao Bang province where there is an important cattle market. The cattle population from the Cao Bang province is considered to belong to the Vietnamese Yellow Cattle breed [31], which is of small size and is mainly used for meat production. This was in agreement with the observed body measurements showing that Bac-Me cattle are one of the smallest cattle breeds in the Ha Giang province. Therefore, the Bac-Me district could be an important area of introgression of the Yellow cattle breed into the H'Mong cattle population within the Ha Giang province. In the Meo-Vac and Dong-Vang districts, weekly cattle markets occur. Householders from these districts are used to buying their herds from the district market. The Meo-Vac cattle market is considered to be the most important market in the province, and trucks coming from other provinces can reach it. In fact, the main road comes from the Cao Bang province. Therefore, despite a lack of accurate information on the extent of importation of cattle from Cao Bang in the Meo-Vac market, our genetic and morphometric results are in favour of the assumption of admixture between the H'Mong and Yellow cattle. Thus, market practices have acted as a melting pot leading to a mean admixture coefficient lower (0.72) than that in the Bac-Me district (0.82).

In the southwestern districts such as Quan-Ba, Quang-Binh, Hoan Su Phi and Xin-Man, no large cattle market is established. Householders preferentially buy animals in the village or in the commune market. Thus admixture is lower and districts belong to cluster 1 with a mean probability higher than 0.6. However, 75% of the cattle from commune 179 belong to cluster 2 and none to cluster 1 with a probability higher than 0.8. Interviews on the origins show that 66.7% of cattle were given by a Development Project which bought them in the northeastern districts. Such information was in accordance with our observations of admixture rate.

### Management considerations

As pointed out by Taberlet *et al. *[34], domestic species could be considered as endangered from a genetic point of view. It is then important to take measures that promote management of the animal genetic resources. Many studies have evaluated conservation policies and prioritisation of breeds compared within each other [35-37]. The purposes and ways of *in situ *conservation programmes for farm animals are multi-fold (see, for example, FAO [38]) and generally include (1) providing a clear view of the status of the breed, through census of animals and survey of farmers, (2) minimising the rate of inbreeding, through practices such as rotational breeding, and (3) providing economic conditions for the development of the breed. If the population size is large enough and if the amount of within-population genetic variability is not critical, then, in addition, a weak selection could be practised in order to improve some traits of interest. The H'Mong cattle seems to be taller than the Yellow cattle breed, with height values ranging from 104 to 110.5 cm in males and from 102 to 104 cm in females [39]. The H'Mong cattle are smaller than the Chau Doc breed (128 cm for males and 104 cm for females) but of the same size of the Ba Ria and Lai Sin breeds that have been crossbred with the Red Sindhi breed from India [39]. Also within the province, *HW *and *I*_*BL *_contributed in a significant way to the district population's differentiation. It seems that householders from the Ha Giang province prefer taller cattle with compact body size for draught power in the rocky mountains than householders from less mountainous provinces like Cao Bang. This kind of consideration about the required morphology of draft cattle was reported in another tropical country, namely Kenya [40]. Therefore, breeding programmes could focus on increasing the size of the animals, but also need to manage genetic diversity. Genetic diversity of the H'Mong cattle population was found to be on the range reported in the Roslin database. According to Caballero & Toro [20], coancestry is useful to estimate the contribution of each population in order to create a pool of sires. Cattle from the Quan-Ba district should contribute to 70% and the Yen-Minh cattle population to 27%. By conserving such a cattle population, one would be able to conserve a "snapshot" of the genetic diversity from the current H'Mong cattle population. However, if the conservation programme objective is to specifically conserve the original H'Mong breed, it would be interesting to compare this local population with cattle populations from bordering provinces. This would allow to better quantify the gene flow from the Yellow cattle to the H'Mong cattle and thus to delineate populations with the lower influence of Yellow cattle genes.

## Conclusions

One of the interests of our study was the very fine-scale spatial analysis across the area where the breed is raised. Surely it is interesting to study breed relationships. But studying local breeds requires a different approach than selected breeds which have very strict selection schemes. Establishing conservation policies on a local breed without knowing the exact genetic structure may not be very efficient. With fine-scale sampling, we were able to establish a fine-admixture grid of the Ha Giang province that will allow carefully selecting sires for the conservation project. Also, we show the usefulness of combining householder's interviews for (1) understanding the genetic patchwork and, (2) identifying which farming practices influence genetic structure. All this information linked together will allow establishing more sustainable management policies.

## Authors' contributions

NVT and HTH carried out sample collection; LPD did the DNA extraction, PCR and data analysis; NB did the sequencing, BB participated in the laboratory protocols and manuscript revision; CB carried out sample collection, sequencing, the computational analysis and prepared the manuscript; GL and DL participated in the computational analysis; XR participated in the computational analysis and preparation of the manuscript; EV participated in the design of the study and the revision of the manuscript; VCC and VND participated in the coordination of the study; JCM participated in the design, coordination of the study, and revision of the manuscript. All authors have read and approved the final manuscript.

## Supplementary Material

Additional file 1 **Detailed methodology**. More precise information about molecular markers and statistical analysis is given. Click here for file

Additional file 2 **Summary of sampling site characteristics**. For each commune sampled, characteristics about the number of sampled villages, animals, and inhabitants are given. Click here for file

Additional file 3 **Sample information and genetic diversity of cattle populations analysed in this study**. Accession numbers of animals analysed in this study and references. Click here for file

Additional file 4 **Summary of loci and their polymorphism measures**. H_Exp _Roslin: range record in the Roslin database; A: number of alleles, H_Exp_: unbiased expected heterozygosity, H_Obs_: observed heterozygosity, Dis_HWE_: number of district populations deviated from HWE equilibrium after Bonferroni correction, F_ST_. Click here for file

Additional file 5 **Summary of body traits for District populations**. *HW*: height at withers, IGs: index slenderness, *EL*: ear length, *I*_*EL*_*: EL/HW, BL*: body length, *I*_*BL*_*:BL/HW, HG*: heart girth, *I*_*HG*_*: HG/HW*. More precise information about molecular markers and statistical analysis is given. Click here for file

Additional file 6 **Estimated proportion of admixture (*q*_*i*_) of the 25 commune samples in each of the two inferred clusters**. Averaged (*q*_*i*_) values per communes, percentage of animals with *q *values > 0.5 and >0.8 for each of the two inferred clusters. Click here for file

Additional file 7 **Summary of p-values**. Summary of p-values for variables and co-variables on body traits and their average for cluster 1 (South-West) and the cluster (North-East) obtained with STRUCTURE software. Click here for file

Additional file 8 **Minimum spanning network among Vietnamese haplotypes.** Circle areas are proportional to haplotype frequencies. Animals from cluster 1 are represented by light colour and animals from cluster 2 by dark colour. (T) = taurus mtDNA lineage, (Z) = indicus mtDNA lineage. Proportion of taurine and zebu haplotypes found in the two Vietnamese subpopulations. Click here for file

Additional file 9 **% of cattle origins per commune obtained from 814 pedigree-origin by the interviewing of 684 householders**. Percentage of cattle origins per commune with 8 categories defined as follows: Farm (i.e. the animal was born on the farm where the dam and granddame were raised, bulls are rarely known); Village (another farm within the village); Commune; District; District Market (the animal was bought at the district market, with no information about its farm origin); Other district; Outside the Province. Click here for file
