# Supplementary material for: Revealing fine scale subpopulation structure in the Vietnamese H'mong cattle breed for conservation purposes
Source: BMC Genet. 2010 Jun 7;11:45. doi: 10.1186/1471-2156-11-45 (PMC2889845; doi:10.1186/1471-2156-11-45)
Supplement: Additional file 1 — Detailed methodology. More precise information about molecular markers and statistical analysis is given. [file 1471-2156-11-45-S1.DOC]

**Additional file 1**

**Material and methods**

**Material sampling :**

See summary of sampling sites characteristics in Table S1

**Methods**

***Molecular methods***

*Monoparental markers*

Kikkawa *et al. (*2003) showed that a unique SNP at position 641 of the *SRY* gene allows a strict differentiation between *Bos indicus* (T) and *B. taurus* (G) origins. Genotyping of this SNP was conducted by pyrosequencing: a 67 bp SRY target was amplified using standard PCR conditions with primers SRY-F1 (5’-CATTGGCTACACGGATTTCG-3’) and SRY-R1 (5’-GCACAAGAAAGTCCAGGCTCTA-3’, biotinylated). Genotypes were determined by sequencing by synthesis on PyroMark Q24 (Biotage), with sequencing primer SRY-S1 (5’-CGGCGGACTTTCCCT-3’) and nucleotide dispensation CGTACAGC. Within the Ha Giang samples, all the bulls (145) were analysed and samples of Gascon taurine breed (3) and Arab zebu breed (3) from Tchad were used as controls.

The complete mtDNA D-loop region was amplified on the 145 bulls using standard PCR conditions with primers L15737 (5’-CTGCAGTCTCACCATCAACC-3’) and
H992 (5’-GATTATAGAACAGGCTCCTC-3’) (Loftus *et al.*, 1994). PCR reactions were sent to MGW company for sequencing using L15737 primer.

Sequences from GenBank from 23 breeds were used and organised in four groups (Additional file 3): European taurine group (Montbéliarde and Simmental), Chinese taurine group (Tongjiang taurine, Anxi, Ebian, Hanyuan, Yanbian), Chinese zebu group (Tongjiang zebu, Ji’an, Leizhou) and Chinese admixed group (Sajiang, Bashan, Bohai, Huangpi, Jiaxian, Jinnan, Luxi, Nanyang, Qinchuan, Wanan, WXizhen, Yunnan YC, Zaobei). The cattle mtDNAs that newly sequenced in this study have been deposited in GenBank under accession numbers FJ800840-FJ800962.

*Biparental markers: microsatellite markers*

Genotypes were done in the laboratory from the National Institute of Animal Husbandry in Hanoi. The thirty microsatellites of the Secondary FAO Guidelines organised in 6 multiplexes were used. The PCR products were labelled with fluorescent dyes coupled to primers and genotyped using a capillary sequencer (Beckman Coulter CEQ8000). However, technical difficulties were encountered for 5 markers. Therefore, genetic analysis was performed on 25 markers only: BM1818, BM1824, BM2113, ETH10, ETH152, ETH185, ETH225, ETH3, HAUT2, HEL1, HEL13, HEL5, HEL9, ILSTS005, ILSTS006, INRA005, INRA023, INRA035, INRA37, INRA063, MM12, SPSS115, TCLA122, TGLA126, TGLA227 (Additional file 4). Thirty control samples from taurine breeds (Gascon and Salers) and African zebu were used for calibration to be able to combine with breed genotypes available at the Cattle Diversity Database (http://www.projects.roslin.ac.uk/cdiv/). From this database, genotypes for 8 markers (BM1818, ETH3, INRA063, HEL1, TGLA227, TGLA122, BM2113, INRA023) were found to be in common with 7 relevant breeds: 2 Indian zebu breeds (Nellore and Ongole), 3 Asian taurine breeds (Japanese Black, Hanwoo and Chinese Yellow cattle) and 2 European taurine (Simmental and Holstein) (Table 1). Genotypes of the H’mong breed are available upon request.

***Statistical analysis***

###### Morphometric Analysis

Morphological data were analysed using single trait linear hierarchical mixed models with the SAS® software. For a given trait, the model was:

*y_ijkl_* = μ + *b* x *alt_ijkl_* + *s_i_* + *d_j_* + *c_ijk_* +*e_ijkl_*

where *y* is the observation, μ the overall mean, *alt* the altitude where the animal is raised and *b* the coefficient of regression of the performance over altitude, *s_i_* the fixed effect of sex *i* (*i* = 1 or 2), *d_j_* the fixed effect of district *j*, *c_ijk_* the random effect of the commune nested into district and *e_ijkl_* a random error. Afterwards, instead of considering district populations, we used cluster populations obtained from the Bayesian approach. The random effect of the district nested into the cluster was added to the model. For some measures, transformations were needed in order to obtain normality and homoskedasticity of model residuals. The P-values of pairwise mean adjusted comparisons were corrected with sequential Bonferroni correction.

A linear discriminant analysis was used on the whole set of markers and measurements. Since this set contains a mix of quantitative and qualitative variables, we used an approach similar to that of Hill & Smith (1976). It is a combination of an internal correspondence analysis on markers (Cazes *et al.* 1998; Laloë *et al.* 2002) and of a principal component analysis on quantitative measures. This approach is based on functions available in the ade4 package (Chessel *et al.* 2004; Dray & Dufour 2007) of the R software (R development core team, 2007).
