# Supplementary material for: Revealing fine scale subpopulation structure in the Vietnamese H'mong cattle breed for conservation purposes
Source: BMC Genet. 2010 Jun 7;11:45. doi: 10.1186/1471-2156-11-45 (PMC2889845; doi:10.1186/1471-2156-11-45)
Supplement: Additional file 2 — Summary of sampling site characteristics. For each commune sampled, characteristics about the number of sampled villages, animals, and inhabitants are given. [file 1471-2156-11-45-S2.DOC]

**Additional file 2**. **Summary of sampling sites characteristics**

| Districts (code) | Commune (code ^a^) | Nv s | Ni | Main Ethnic group | Altitudes range (m) | Mean herd size^b^ |
| --- | --- | --- | --- | --- | --- | --- |
| Quang-Binh (QBn) | Tan-Nam (157) (157) | 4 | 5 | Tay | 150-350 | 2.8 |
|  | Xuan-Giang (179) | 2 | 4 | Tay | 116-210 | 2.7 |
| Hoang Su Phi (HSP) | Po-Lo (85) | 3 | 9 | H'mong | 642-1057 | 1.9 |
|  | Po Ly Ngai (110) | 4 | 13 | Nung | 825-914 | 2.0 |
|  | San Sa Ho (114) | 4 | 12 | Nung/Tay | 646-731 | 1.6 |
| Xin-Man (XM) | Nan-Xin (91) | 3 | 6 | La Chi | 888-1395 | 3.8 |
|  | Chi-Ca (103) | 8 | 45 | H'mong | 950-1410 | 2.5 |
| Quan-Ba (QB) | Bat Dai Son (30) | 6 | 19 | H'mong | 606-1105 | 2.4 |
|  | Tung-Vai (49) | 6 | 11 | H'mong | 890-1010 | 2.6 |
|  | Quet-Tien (56) | 5 | 12 | H'mong | 689-936 | 1.9 |
|  | Lung-Tam (188) | 5 | 12 | H'mong | 429-975 | 2.7 |
| Yen-Minh (YM) | Na-Khe (25) | 6 | 14 | Dao | 457-806 | 1.7 |
|  | Lung-Ho (61) | 7 | 23 | H'mong | 486-756 | 3.1 |
|  | Du-Gia (65) | 4 | 15 | H'mong | 395-916 | 3.1 |
| Dong-Van (DV) | Lun-Pu (1) | 7 | 25 | H'mong | 1288-1427 | 3.5 |
|  | Tai Phin Tung (7) | 6 | 24 | H'mong | 1095-1363 | 2.2 |
|  | Pho-Cao (16) | 8 | 18 | H'mong | 1148-1530 | 2.4 |
|  | San-Tung (19) | 6 | 21 | H'mong | 1284-1511 | 1.8 |
| Meo-Vac (MV) | Lung-Cu (4) | 7 | 28 | H'mong | 968-1210 | 2.6 |
|  | Tat-Nga (40) | 5 | 14 | H'mong | 375-799 | 2.7 |
|  | Nam-Ban (45) | 3 | 8 | Giay | 312-946 | 3.0 |
|  | Khau-Vai (48) | 8 | 32 | H'mong | 461-1245 | 2.6 |
| Bac-Me (BM) | Giap-Trung (75) | 3 | 4 | Dao | 127-678 | 2.9 |
|  | Yen-Phong (89) | 6 | 19 | Tay | 171-445 | 4.3 |
|  | Yen-Cuong (113) | 7 | 9 | H'mong/Dao | 132-392 | 3.3 |

^a^ Number code of communes have been randomly gived by MAPINFO software for cartographic purposes; ^b^ mean householder herd size; Nv: number of villages sampled; Ni: sample size;
