# Supplementary material for: Revealing fine scale subpopulation structure in the Vietnamese H'mong cattle breed for conservation purposes
Source: BMC Genet. 2010 Jun 7;11:45. doi: 10.1186/1471-2156-11-45 (PMC2889845; doi:10.1186/1471-2156-11-45)
Supplement: Additional file 3 — Sample information and genetic diversity of cattle populations analysed in this study. Accession numbers of animals analysed in this study and references. [file 1471-2156-11-45-S3.DOC]

**Additional file 3. Sample information and genetic diversity of cattle populations analyzed in this study**

| Group names | Breed names | Nb ind. | Accession No. | Sources |
| --- | --- | --- | --- | --- |
| European taurine |  |  |  |  |
|  | Montbéliard | 4 | AY521103-6 | Lai et al. 2006 |
|  | Simmental | 4 | AY521118; AY521117,AY521119-120 | Lai et al. 2006 |
| Chinese Taurine |  |  |  |  |
|  | Tongjiang | 38 | EF417933-69 | Chen et al. 2008 |
|  | Anxi | 10 | AY521076-82, AY902382-4 | Lai et al. 2006 |
|  | Ebian | 5 | AY521088-9, AY902387-9 | Lai et al. 2006 |
|  | Hanyuan | 4 | AY521090-3 | Lai et al. 2006 |
|  | Yanbian | 5 | DQ344923-7 | Lei et al. 2006 |
| Chinese Zebu |  |  |  |  |
|  | Tongjiang | 17 | EF417970-86 | Chen et al. 2008 |
|  | Ji'an | 5 | DQ344933-7 | Lei et al. 2006 |
|  | Leizhou | 4 | DQ344965-8 | Lei et al. 2006 |
| Chinese admixed |  |  |  |  |
|  | Sajiang | 7 | AY521112-6, AY902396-7 | Lai et al. 2006 |
|  | Bashan | 7 | AY521083-7, AY902385-6 | Lai et al. 2006 |
|  | Bohai | 5 | DQ344928-32 | Lei et al. 2006 |
|  | Huangpi | 4 | AY521096-8, AY902393 | Lai et al. 2006 |
|  | Jiaxian | 5 | AY119667-8, AY119672; DQ344944-5 | Lai et al. 2006, Lei et al. 2006 |
|  | Jinnan | 8 | AY521100-2, AY902394, AY119669, AY119676; DQ344942-43 | Lei et al. 2004, Lai et al. 2006, Lei et al. 2006 |
|  | Luxi | 5 | DQ344946-50 | Lei et al. 2006 |
| . | Nanyang | 5 | AY115525-7;DQ344957-8 | Lei et al. 2004,Lei et al 2006 |
|  | Qinchuan | 12 | AY521107-11, AY902395, AF514784, AY119673-4; DQ344959-61 | Lei et al. 2004, Lai et al. 2006, Lei et al. 2006 |
|  | Wannan | 6 | AY521121-6, AY902398-9 | Lai et al. 2006 |
|  | Xizhen | 5 | AY119670-1;DQ344969-70 | Lai et al. 2006, Lei et al. 2006 |
|  | Yunnan YC | 7 | AY521127-9, AY902400-3 | Lai et al. 2006 |
|  | Zaobei | 9 | AY521130-36, AY902404-5 | Lai et al. 2006 |

**References cited in Table S2:**

Chen S.Y., Liu Y.P., Wang W., Gao C.Z., Yai Y.G., Lai S.J. (2008) Dissecting the matrilineal

component of the Tongjiang cattle from Southwest China. *Biochemical Genetics,* **46,** 206-15

Lai S.J., Liu Y.P., Liu Y.X., Li X.W.. (2006) Genetic diversity and origin of the Chinese cattle revealed by mtDNA D-loop sequence variation. *Molecular Phylogenetics and Evolution,* **38,** 146-54

Lei C.Z., Chen H., Yang G.S., Song L.S., Lei X.Q., Sun W.B., Li R.B., Liu X.L. (2004)

Study on Mitochondrial DNA Genetic Diversity of Some Cattle Breeds in China. *Acta*

*Genetica Sinica*, **31,** 57-62. (In Chinese)

Lei C.Z., Chen H., Zhang H.C., Cai X., Liu R.Y., Luo L.Y.? Wang C.F., Zhang W., Ge Q.L., Zhang R.F., Lan X.Y., Sun W.B. (2006) Origin and phylogeographical structure of Chinese cattle. *Animal Genetics*, **37**, 579-82.
