# Supplementary material for: Revealing fine scale subpopulation structure in the Vietnamese H'mong cattle breed for conservation purposes
Source: BMC Genet. 2010 Jun 7;11:45. doi: 10.1186/1471-2156-11-45 (PMC2889845; doi:10.1186/1471-2156-11-45)
Supplement: Additional file 4 — Summary of loci and their polymorphism measures. HExp Roslin: range record in the Roslin database; A: number of alleles, HExp: unbiased expected heterozygosity, HObs: observed heterozygosity, DisHWE: number of district populations deviated from HWE equilibrium after Bonferroni correction, FST. [file 1471-2156-11-45-S4.DOC]

**Additional file 4**.**Summary of loci and their polymorphism measures**: HExp Roslin: range record in Roslin database; A: number of alleles, HExp: unbiased expected heterozygosity, HObs: observed heterozygosity, DisHWE: number of district populations deviated from HWE equilibrium after Bonferroni correction, FST

| Loci | Range | H_Exp_ Roslin | A | H_Exp_ | H_Obs_ | Dis_HWE_ | F_ST_ |
| --- | --- | --- | --- | --- | --- | --- | --- |
| BM1818 | 256-270 | 0.270-0.826 | 7 | 0.760 | 0.708 | 0 | 0.004 |
| BM1824 | 178-194 | 0.441-0.875 | 6 | 0.690 | 0.657 | 0 | 0.019 |
| BM2113 | 122-140 | 0.205-0.901 | 8 | 0.700 | 0.699 | 0 | 0.000 |
| ETH10 | 208-220 | 0.160-0.809 | 7 | 0.765 | 0.750 | 0 | 0.003 |
| ETH152 | 190-202 | 0.210-0.836 | 7 | 0.550 | 0.537 | 0 | 0.007 |
| ETH185 | 222-240 | 0.583-0.813 | 7 | 0.766 | 0.456 | 6 | 0.023 |
| ETH225 | 136-164 | 0.362-0.842 | 9 | 0.848 | 0.795 | 1 | 0.009 |
| ETH3 | 111-125 | 0.465-0.811 | 6 | 0.576 | 0.542 | 0 | 0.035 |
| HAUT27 | 127-151 | 0.406-0.812 | 10 | 0.846 | 0.821 | 0 | 0.010 |
| HEL1 | 99-109 | 0.402-0.800 | 5 | 0.443 | 0.293 | 3 | 0.032 |
| HEL13 | 181-191 | 0.279-0.801 | 6 | 0.696 | 0.512 | 5 | 0.019 |
| HEL5 | 149-165 | 0.459-0.873 | 8 | 0.793 | 0.396 | 5 | 0.018 |
| HEL9 | 140-166 | 0.210-0.883 | 13 | 0.868 | 0.815 | 0 | 0.005 |
| ILSTS005 | 176-192 | 0.142-0.745 | 7 | 0.508 | 0.431 | 1 | 0.007 |
| ILSTS006 | 272-302 | 0.436-0.807 | 13 | 0.821 | 0.755 | 1 | 0.005 |
| INRA005 | 133-145 | 0.442-0.747 | 6 | 0.786 | 0.749 | 0 | 0.008 |
| INRA023 | 195-215 | 0.478-0.897 | 7 | 0.751 | 0.707 | 0 | 0.022 |
| INRA035 | 101-123 | 0.111-0.790 | 6 | 0.768 | 0.693 | 0 | 0.005 |
| INRA037 | 113-133 | 0.524-0.820 | 9 | 0.591 | 0.583 | 0 | 0.005 |
| INRA063 | 170-184 | 0.157-0.806 | 5 | 0.696 | 0.698 | 0 | 0.008 |
| MM12 | 106-122 | 0.534-0.862 | 8 | 0.770 | 0.690 | 0 | 0.014 |
| SPSS115 | 243-259 | 0.357-0.854 | 9 | 0.835 | 0.751 | 2 | 0.018 |
| TGLA122 | 137-167 | 0.265-0.869 | 12 | 0.821 | 0.799 | 0 | 0.010 |
| TGLA126 | 117-127 | 0.418-0.804 | 6 | 0.707 | 0.632 | 0 | 0.010 |
| TGLA227 | 72-96 | 0.100-0.905 | 8 | 0.648 | 0.620 | 0 | 0.004 |
