# Supplementary material for: Revealing fine scale subpopulation structure in the Vietnamese H'mong cattle breed for conservation purposes
Source: BMC Genet. 2010 Jun 7;11:45. doi: 10.1186/1471-2156-11-45 (PMC2889845; doi:10.1186/1471-2156-11-45)
Supplement: Additional file 5 — Summary of body traits for District populations. HW: height at withers, IGs: index slenderness, EL: ear length, IEL: EL/HW, BL: body length, IBL:BL/HW, HG: heart girth, IHG: HG/HW. More precise information about molecular markers and statistical analysis is given. [file 1471-2156-11-45-S5.DOC]

***Additional file 5. Summary of body traits for District populations.*** *HW: height at withers, IGs: index slenderness, EL: ear length, I_EL_: EL/HW, BL: body length, I_BL_:BL/HW, HG: heart girth, I_HG_*: HG/HW.

| Sexe | District | HW | IG_s_ | EL | I_EL_ | BL | I_BL_ | HG | I_HG_ |
| --- | --- | --- | --- | --- | --- | --- | --- | --- | --- |
| Female | HSP | 112.0±6.8 | 0.85±0.09 | 18.6±1.6 | *0.17±0.01* | 103.6±8.1 | *0.93±0.07* | 138.6±9.3 | *1.25±0.07* |
|  | XM | **115.5±4.9** | 0.82±0.09 | **19.4±1.8** | 0.17±0.02 | 107.7±7.3 | 0.94±0.07 | **144.1±7.5** | 1.25±0.05 |
|  | QB | 111.6±5.3 | *0.80±0.08* | 19.2±2.2 | 0.17±0.02 | 105.8±6.7 | 0.95±0.06 | 142.4±6.8 | 1.28±0.07 |
|  | YM | 107.9±5.4 | 0.84±0.11 | 19.2±2.2 | **0.18±0.02** | **107.9±7.8** | **1.01±0.06** | 139.7±8.2 | **1.30±0.06** |
|  | DV | 109.0±6.6 | 0.86±0.11 | 19.2±2.1 | 0.18±0.02 | 106.7±8.8 | 0.99±0.07 | 141.0±9.4 | 1.29±0.06 |
|  | MV | *106.0±5.8* | **0.88±0.09** | 18.9±2.5 | **0.18±0.02** | 102.3±8.3 | 0.97±0.08 | *134.2±6.7* | 1.27±0.06 |
|  | BM | 106.0±5.8 | 0.79±0.10 | *18.4±1.8* | 0.17±0.02 | *99.9±7.6* | 0.94±0.06 | 136.8±8.4 | 1.29±0.06 |
| Male | HSP | 118.6±4.5 | 0.81±0.07 | 18.8±1.4 | 0.16±0.01 | 105.8±9.0 | *0.89±0.06* | 147.0±7.9 | *1.24±0.05* |
|  | XM | **121.8±8.9** | *0.77±0.11* | **19.6±1.6** | 0.16±0.01 | 111.6±12.3 | 0.92±0.07 | 150.8±15.6 | 1.25±0.05 |
|  | QB | 119.0±7.7 | 0.77±0.08 | 19.2±2.7 | 0.16±0.02 | **111.9±9.5** | 0.95±0.06 | **155.0±13.8** | 1.30±0.08 |
|  | YM | 116.0±8.2 | 0.80±0.13 | 19.3±2.4 | 0.17±0.02 | 108.0±10.4 | **0.99±0.08** | 146.1±16.7 | 1.28±0.10 |
|  | DV | 114.1±7.3 | **0.84±0.11** | 18.8±1.9 | 0.16±0.01 | 109.0±9.5 | 0.96±0.07 | 145.6±14.9 | 1.28±0.07 |
|  | MV | *111.6±8.6* | 0.84±0.11 | 19.0±2.1 | 0.17±0.02 | 104.1±10.3 | 0.95±0.09 | *142.4±15.0* | 1.27±0.07 |
|  | BM | 114.1±9.4 | 0.74±0.12 | *17.3±1.4* | *0.15±0.01* | *105.2±9.7* | 0.92±0.06 | 147.5±16.5 | **1.29±0.08** |

**in bold**: maximum values, *in italic*: minimum values
