# Supplementary material for: Revealing fine scale subpopulation structure in the Vietnamese H'mong cattle breed for conservation purposes
Source: BMC Genet. 2010 Jun 7;11:45. doi: 10.1186/1471-2156-11-45 (PMC2889845; doi:10.1186/1471-2156-11-45)
Supplement: Additional file 6 — Estimated proportion of admixture (qi) of the 25 commune samples in each of the two inferred clusters. Averaged (qi) values per communes, percentage of animals with q values > 0.5 and >0.8 for each of the two inferred clusters. [file 1471-2156-11-45-S6.DOC]

**Additional file 6. Estimated proportion of admixture (*q*_i_) of the 25 commune samples in each of the two inferred clusters**

| District | Commune | N | Mean *q*(1) | Mean *q*(2) | % *q*(1) >0.5 | % *q*(2) >0.5 | % *q*(1) >0.8 | % *q*(2) >0.8 |
| --- | --- | --- | --- | --- | --- | --- | --- | --- |
| BM |  | 34 | 0.18 | **0.82** | 5.9 | **94.1** | 0.0 | **73.5** |
|  | 75 | 4 | 0.41 | **0.59** | 25.0 | **75.0** | 0.0 | **25.0** |
|  | 89 | 19 | 0.14 | **0.86** | 0.0 | **100.0** | 0.0 | **78.9** |
|  | 113 | 9 | 0.12 | **0.88** | 0.0 | **100.0** | 0.0 | **88.9** |
| MV |  | 82 | 0.28 | **0.72** | 15.9 | 84.1 | 2.4 | 42.7 |
|  | 4 | 28 | 0.28 | **0.72** | 17.9 | **82.1** | 0.0 | **46.4** |
|  | 40 | 14 | 0.31 | **0.69** | 21.4 | **78.6** | 7.1 | **35.7** |
|  | 45 | 8 | 0.26 | **0.74** | 0.0 | **100.0** | 0.0 | **25.0** |
|  | 48 | 32 | 0.28 | **0.72** | 15.6 | **84.4** | 3.1 | **46.9** |
| DV |  | 88 | 0.40 | **0.60** | 36.4 | **63**.6 | 4.5 | **30.7** |
|  | 1 | 25 | 0.42 | **0.58** | 40.0 | **60.0** | 4.0 | **28.0** |
|  | 7 | 24 | 0.35 | **0.65** | 29.2 | **70.8** | 0.0 | **41.7** |
|  | 16 | 18 | 0.48 | **0.52** | **55.6** | 44.4 | 16.7 | **22.2** |
|  | 19 | 21 | 0.34 | **0.66** | 23.8 | **76.2** | 0.0 | **28.6** |
| YM |  | 52 | 0.46 | **0.54** | 44.2 | **55.8** | 9.6 | **21.2** |
|  | 25 | 14 | **0.58** | 0.42 | **64.3** | 35.7 | 21.4 | 14.3 |
|  | 61 | 23 | 0.38 | **0.62** | 34.8 | **65.2** | 0.0 | **34.8** |
|  | 65 | 15 | 0.47 | **0.53** | 40.0 | **60.0** | **13.3** | 6.7 |
| QB |  | 54 | **0.68** | 0.32 | **79.6** | 20.4 | **35.2** | 3.7 |
|  | 30 | 19 | **0.74** | 0.26 | **89.5** | 10.5 | **36.8** | 0.0 |
|  | 49 | 11 | **0.80** | 0.20 | **100.0** | 0.0 | **63.6** | 0.0 |
|  | 56 | 12 | **0.51** | 0.49 | 50.0 | 50.0 | 8.3 | 8.3 |
|  | 188 | 12 | **0.66** | 0.34 | **75.0** | 25.0 | **33.3** | 8.3 |
| QBn |  | 9 | **0.60** | 0.40 | **66.7** | 33.3 | 44.4 | 33.3 |
|  | 157 | 5 | **0.86** | 0.14 | **100.0** | 0.0 | **80.0** | 0.0 |
|  | 179 | 4 | 0.27 | **0.73** | 25.0 | **75.0** | 0.0 | **75.0** |
| HSP |  | 34 | **0.77** | 0.23 | **91.2** | 8.8 | **61.8** | 2.9 |
|  | 85 | 9 | **0.63** | 0.37 | **77.8** | 22.2 | **33.3** | 0.0 |
|  | 110 | 13 | **0.78** | 0.22 | **92.3** | 7.7 | **69.2** | 7.7 |
|  | 114 | 12 | **0.86** | 0.14 | **100.0** | 0.0 | **75.0** | 0.0 |
| XM |  | 54 | **0.83** | 0.17 | **94.4** | 5.6 | **68.5** | 0.0 |
|  | 91 | 6 | **0.72** | 0.28 | **83.3** | 16.7 | **50.0** | 0.0 |
|  | 103 | 45 | **0.84** | 0.16 | **95.6** | 4.4 | **68.9** | 0.0 |

**in bold:** highest mean *q*_i_ values; N: number of individuals, % (*q*_i_) > X: percentage of animals with *q*-value for cluster *i* > X.
