# Supplementary material for: Revealing fine scale subpopulation structure in the Vietnamese H'mong cattle breed for conservation purposes
Source: BMC Genet. 2010 Jun 7;11:45. doi: 10.1186/1471-2156-11-45 (PMC2889845; doi:10.1186/1471-2156-11-45)
Supplement: Additional file 7 — Summary of p-values. Summary of p-values for variables and co-variables on body traits and their average for cluster 1 (South-West) and the cluster (North-East) obtained with STRUCTURE software. [file 1471-2156-11-45-S7.DOC]

**Additional file 7**. **Summary of p-values for variables and co-variables on body traits and their average for cluster 1 (South-West) and the cluster (North-East) obtained with STRUCTURE software.**

| Body trait | Transformation | Sexe | Altitude | Cluster | Male | | Female | |
| --- | --- | --- | --- | --- | --- | --- | --- | --- |
|  |  |  |  |  | South-West | North-East | South-West | North-East |
| HW | none | *<0.0001* | 0,1594 | *0,0352* | 119.0±8.1 | 113.6 ± 8.5 | 112.7±6 | 107.6±6.2 |
| IGs | Racine | *<0.0002* | 0,1356 | 0,5269 | 0.78±0.09 | 0.82±0.12 | 0.82±0.09 | 0.85±0.11 |
| BL | Ln | *0,0031* | 0,5372 | 0,8514 | 109.9±11.5 | 108.9±12.5 | 106.2±8.3 | 104.9±9.5 |
| I_BL_ | Racine | *<0.0002* | 0,8255 | 0,1718 | 0.92±0.07 | 0.95±0.07 | 0.94±0.07 | 0.98±0.07 |
| HG | none | *<0.0001* | *0,0109* | 0,5219 | 152.4±12.9 | 144.6±14.1 | 141.4±8.3 | 138.4±9 |
| I_HG_ | racine | *<0.0001* | *0,0437* | 0,0923 | 1.27±0.07 | 1.28±0.08 | 1.26±0.07 | 1.29±0.06 |
| EL | none | 0,2956 | 0,3236 | 0,3867 | 19±2.1 | 18.8±2 | 19.2±1.9 | 18.9±2.2 |
| I_EL_ | none | *<0.0001* | 0,9722 | 0,9722 | 0.16±0.02 | 0.17±0.02 | 0.17±0.02 | 0.18±0.02 |

*in italic*: significant p-values
