# Supplementary material for: Revealing fine scale subpopulation structure in the Vietnamese H'mong cattle breed for conservation purposes
Source: BMC Genet. 2010 Jun 7;11:45. doi: 10.1186/1471-2156-11-45 (PMC2889845; doi:10.1186/1471-2156-11-45)

**Additional file 8. Minimum spanning tree among Vietnamese haplotypes. Circle areas are proportional to haplotype frequencies. Animals from cluster 1 are represented by light colour and animals from cluster 2 by dark colour. (T) = taurus mtDNA lineage, (Z) = indicus mtDNA lineage.**


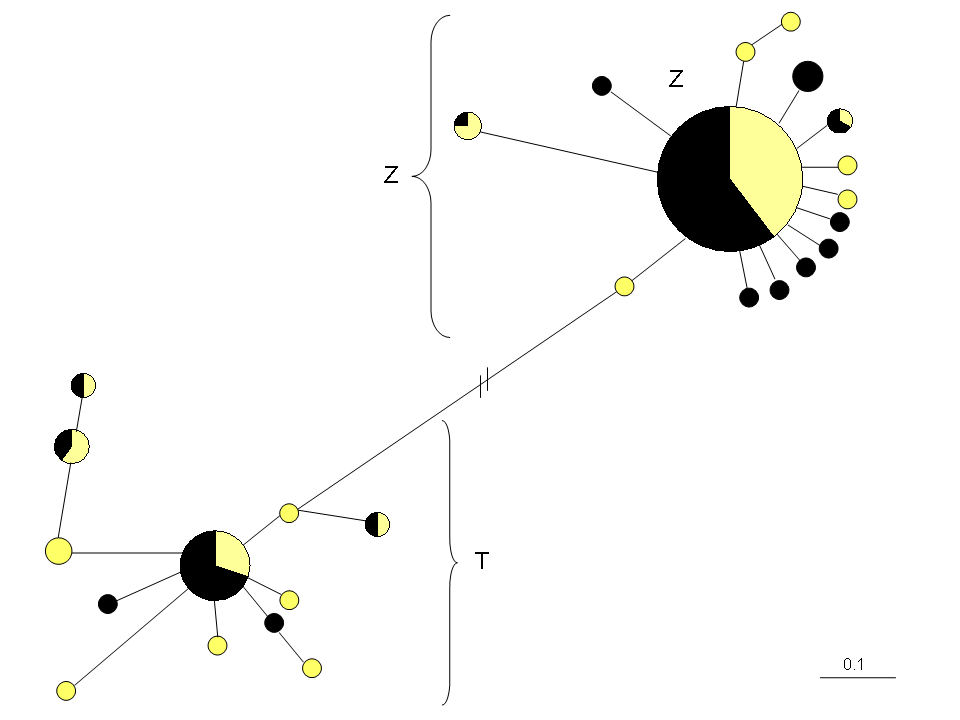

Supplement: Additional file 8 — Minimum spanning network among Vietnamese haplotypes. Circle areas are proportional to haplotype frequencies. Animals from cluster 1 are represented by light colour and animals from cluster 2 by dark colour. (T) = taurus mtDNA lineage, (Z) = indicus mtDNA lineage. Proportion of taurine and zebu haplotypes found in the two Vietnamese subpopulations. [file 1471-2156-11-45-S8.DOC]
