# Supplementary material for: Revealing fine scale subpopulation structure in the Vietnamese H'mong cattle breed for conservation purposes
Source: BMC Genet. 2010 Jun 7;11:45. doi: 10.1186/1471-2156-11-45 (PMC2889845; doi:10.1186/1471-2156-11-45)
Supplement: Additional file 9 — % of cattle origins per commune obtained from 814 pedigree-origin by the interviewing of 684 householders. Percentage of cattle origins per commune with 8 categories defined as follows: Farm (i.e. the animal was born on the farm where the dam and granddame were raised, bulls are rarely known); Village (another farm within the village); Commune; District; District Market (the animal was bought at the district market, with no information about its farm origin); Other district; Outside the Province. [file 1471-2156-11-45-S9.DOC]

**Additional file 9**. % of cattle origins per commune obtained from 814 pedigree-origin by interviewing of 684 householders

| District | Commune | Family | Village | Commune | District | District Market | Province | Project | Other Province |
| --- | --- | --- | --- | --- | --- | --- | --- | --- | --- |
| *BM* | 75 | 10.0 | 30.0 | 10.0 | 30.0 | 0.0 | 10.0 | 0.0 | 10.0^a^ |
|  | 89 | 12.5 | 37.5 | 10.7 | 10.7 | 0.0 | 3.6 | 1.8 | 23.2^a^ |
|  | 113 | 12.9 | 41.9 | 6.5 | 22.6 | 0.0 | 0.0 | 9.7 | 6.5 ^a^ |
| *MV* | 4 | 16.0 | 6.0 | 2.0 | 12.0 | 64.0 | 0.0 | 0.0 | 0.0 |
|  | 40 | 22.0 | 19.5 | 7.3 | 4.9 | 46.3 | 0.0 | 0.0 | 0.0 |
|  | 45 | 6.7 | 33.3 | 20.0 | 20.0 | 20.0 | 0.0 | 0.0 | 0.0 |
|  | 48 | 19.2 | 7.7 | 32.7 | 23.1 | 17.3 | 0.0 | 0.0 | 0.0 |
| *DV* | 1 | 6.7 | 37.8 | 31.1 | 6.7 | 13.3 | 0.0 | 0.0 | 4.4 ^b^ |
|  | 7 | 0.0 | 22.4 | 10.3 | 31.0 | 36.2 | 0.0 | 0.0 | 0.0 |
|  | 16 | 3.1 | 12.5 | 40.6 | 31.3 | 3.1 | 9.4 | 0.0 | 0.0 |
|  | 19 | 2.2 | 19.6 | 6.5 | 30.2 | 30.4 | 8.7 | 0.0 | 2.2 ^b^ |
| *YM* | 25 | 26.3 | 36.8 | 23.7 | 7.9 | 0.0 | 5.3 | 0.0 | 0.0 |
|  | 61 | 33.3 | 19.3 | 24.6 | 22.8 | 0.0 | 0.0 | 0.0 | 0.0 |
|  | 65 | 46.7 | 13.3 | 20.0 | 20.0 | 0.0 | 0.0 | 0.0 | 0.0 |
| *QB* | 30 | 0.0 | 20.8 | 33.3 | 25.0 | 0.0 | 16.7 | 4.2 | 0.0 |
|  | 49 | 33.3 | 20.0 | 33.3 | 6.7 | 0.0 | 6.7 | 0.0 | 0.0 |
|  | 56 | 4.3 | 30.4 | 26.1 | 34.8 | 0.0 | 4.3 | 0.0 | 0.0 |
|  | 188 | 25.0 | 28.1 | 21.9 | 21.9 | 0.0 | 3.1 | 0.0 | 0.0 |
| *QBn* | 157 | 0.0 | 0.0 | 20.0 | 20.0 | 0.0 | 60.0 | 0.0 | 0.0 |
|  | 179 | 4.8 | 0.0 | 0.0 | 9.5 | 0.0 | 19.0 | 66.7 | 0.0 |
| *HSP* | 85 | 5.0 | 10.0 | 30.0 | 50.0 | 0.0 | 5.0 | 0.0 | 0.0 |
|  | 110 | 6.5 | 41.9 | 16.1 | 9.7 | 6.5 | 6.5 | 12.9 | 0.0 |
|  | 114 | 8.3 | 41.7 | 8.3 | 33.3 | 0.0 | 8.3 | 0.0 | 0.0 |
| *XM* | 91 | 0.0 | 25.0 | 50.0 | 25.0 | 0.0 | 0.0 | 0.0 | 0.0 |
|  | 103 | 4.5 | 39.4 | 27.3 | 22.7 | 0.0 | 1.5 | 0.0 | 4.5 ^b^ |

Farm (i.e. the animal was born on the farm where the dam and granddam were raised, bulls are rarely known); Village (another farm within the village); Commune; District; District Market (the animal was bought at the district market, with no information about its farm origin); Other district; Outside the Province; Project (many poverty alleviation projects have given buffaloes or cattle to householders, therefore a class was made for these “gifts” because their geographical origin was not possible to determine); a: from Cao Bang; b: from China
